# Technologies that promote health education for the community elderly: integrative review

**DOI:** 10.1590/1518-8345.3171.3186

**Published:** 2019-10-14

**Authors:** Guilherme Guarino de Moura Sá, Fernanda Lorrany Silva, Ana Maria Ribeiro dos Santos, Julyanne dos Santos Nolêto, Márcia Teles de Oliveira Gouveia, Lídya Tolstenko Nogueira

**Affiliations:** 1 Universidade Federal do Piauí, Departamento de Enfermagem, Teresina, PI, Brasil.

**Keywords:** Aged, Health of the Elderly, Educational Technology, Health Education, Teaching Materials, Review, Idoso, Saúde do Idoso, Tecnologia Educacional, Educação em Saúde, Materiais de Ensino, Revisão, Anciano, Salud del Anciano, Tecnología Educacional, Educación en Salud, Materiales de Enseñanza, Revisión

## Abstract

**Objective:**

to identify in the scientific literature the technologies developed to promote health education for the community elderly.

**Method:**

integrative review that included original articles indexed by Latin American and Caribbean Literature in Health Sciences, *Medical Literature Analysis and Retrieval System Online*, *Cumulative Index to Nursing and Allied Health Literature*, Scopus, Web of Science, Science Direct, and Cochrane databases, without restriction of time and language. Results were analyzed descriptively, in five analytical categories.

**Results:**

Fifteen articles published on national and international journals were selected, with predominance of experimental studies that tested the effects of such technologies. The types of educational technology developed were printed materials, software and video, as well as mock-up and telephone support. Falls in the elderly were the most discussed theme. The studies have shown that the types of technology found are feasible to promote health education for the community elderly.

**Conclusion:**

The technologies developed to promote health education for the elderly were multiple and proved effective for use in community interventions.

## Abstract (pt)

**Objetivo:**

identificar na literatura científica as tecnologias desenvolvidas para a educação em saúde de idosos na comunidade.

**Método:**

revisão integrativa que incluiu artigos originais indexados nas bases de dados Literatura Latino Americana e do Caribe em Ciências da Saúde, Medical Literature Analysis and Retrieval System Online, Cumulative Index to Nursing and Allied Health Literature, Scopus, Web of Science, Science Direct e Cochrane, sem restrição de tempo e idioma. A análise dos resultados ocorreu na forma descritiva, em cinco categorias analíticas.

**Resultados:**

foram selecionados 15 artigos, publicados em revistas nacionais e internacionais, com predomínio de estudos experimentais que testaram os efeitos das tecnologias. Os tipos de tecnologia educacional desenvolvidos foram material impresso, software e vídeo, além de maquete e suporte telefônico. O tema mais abordado foi a queda. Os estudos mostraram que os tipos de tecnologia encontrados são viáveis para a educação em saúde de idosos na comunidade.

**Conclusão:**

as tecnologias desenvolvidas para a educação em saúde de idosos foram múltiplas e mostraram-se eficazes para serem utilizadas em intervenções na comunidade.

## Abstract (es)

**Objetivo:**

identificar en la literatura científica las tecnologías que fueron desarrolladas para la educación en salud de ancianos en la comunidad.

**Método:**

revisión integrativa que incluyó artículos originales indexados en las bases de datos Literatura Latinoamericana y del Caribe en Ciencias de la Salud, *Medical Literature Analysis and Retrieval System Online*, *Cumulative Index to Nursing and Allied Health Literature*, Scopus, Web of Science, Science Direct y Cochrane, sin restricción de tiempo e idioma. El análisis de los resultados ocurrió de manera descriptiva, en cinco categorías que son analíticas.

**Resultados:**

fueron seleccionados 15 artículos, que fueron publicados en revistas nacionales e internacionales, con predominio de estudios experimentales que probaron los efectos de las tecnologías. Los tipos de tecnología educativa que fueron desarrollados fueron los materiales impresos, como también programa y vídeo, además de maqueta y soporte telefónico. El tema que fue más abordado fue la caída. Los estudios mostraron que los tipos de tecnología que fueron encontrados son viables para la educación en salud de ancianos en la comunidad.

**Conclusión:**

las tecnologías que fueron desarrolladas para la educación en salud de ancianos fueron múltiples y se mostraron eficaces para ser utilizadas en intervenciones en la comunidad.

## Introduction

The increase in the number of elderly people observed worldwide is a milestone in the longevity transformation process and emerges from changes in the behavior of fertility and mortality rates. Globally, this demographic segment represents 12.3% of the population, with a growth rate of 3% per year. Global projections point to increase in the number of people over 60 years old from 1.4 billion in 2030 to 2.1 billion in 2050, a 50% increase in 20 years. Nevertheless, declines in physical and mental capacity, mainly associated with chronic health conditions, accompany demographic and epidemiological changes in the population profile^1^

In this scenario, the demands of health services also increase, especially when considering life multiple dimensions and the heterogeneity of the elderly living in the community^2^. Thus, implementing actions that consider integrality and promote active aging is an important challenge for the elderly health. Therefore, health professionals should optimize health promotion strategies that enhance the social participation of the elderly and respect their autonomy^3-4^.

For this purpose, health education is a necessary tool to promote the elderly health, since it provides knowledge for the prevention and reduction of diseases, makes the person active in the life transformation, and encourages self-care and search for autonomy. With regard to Gerontological Nursing, health education is an integral part of nurses’ clinical practice and allows for creativity and multiplicity of choices. However, it is essential to consider the elderly singularity so that to stimulate changes in individual behavior^5-6^.

In this context, the technical-scientific advance enabled the emergence of educational technologies. They are result of concrete processes, based on daily experiences, and aimed at the methodical development of knowledge and knowing to be used for specific practical purposes. Therefore, use of educational technologies is seen as improvement to care orientation for the community elderly^7-8^.

Due to this reality, educational technologies in the tactile and auditory, expository and dialogical, printed and audiovisual modalities are considered methodological strategies to promote health education for the elderly^9^. Thus, introducing such technologies contributes to the construction of the elderly’s self-care knowledge and empowerment.

However, it should be noted that no review studies that presented the technologies already developed to promote health education for the community elderly were found in a wide search in the national and international literature. In this perspective, this study arises from the need for filling this knowledge gap.

For this reason, the intention is to contribute to the Evidence-Based Practice (PBE) in order to potentate the process of acquiring knowledge and decision making of health professionals, especially nurses, in order for them to choose the best level of evidence to make the educative elderly care operational.

In view of the exposed above, this study was aimed at identifying in the scientific literature the technologies developed to promote health education for the community elderly.

## Method

It is an integrative review, structured in six distinct stages: 1) elaboration of the research question; 2) definition of databases and criteria for study inclusion and exclusion; 3) definition of the information to be extracted from the selected studies; 4) evaluation of the studies included in the review; 5) interpretation of results; 6) presentation of the knowledge review/synthesis^10^.

The study was guided by a protocol developed by the researchers. The research question was elaborated according to the PICo strategy (P − population, I − interest, Co − Context)^11^. The following structure was thus considered: P – the elderly; I – educational technology; Co – health education, and the question elaborated was: “What are the technologies developed to promote health education for the community elderly available in the literature?”

The bibliographic survey was carried out in August 2018, through virtual access to the following databases: Latin American and Caribbean Literature in Health Sciences (LILACS), via consultation with the Virtual Health Library (VHL); *Medical Literature Analysis and Retrieval System Online* (MEDLINE), accessed through the PubMed portal; *Cumulative Index to Nursing and Allied Health Literature* (CINAHL), via Core Collection (Thomson Reuters); Scopus (Elsevier); Web of Science; Science Direct, and Cochrane. In addition, a manual search was also performed by reading the references of the primary studies included.

The inclusion criteria adopted were: primary articles that presented educational technology developed for people aged 60 or older living in the community, published by August 2018, in any language. The exclusion criteria were: editorials, theses, dissertations, review articles already selected when searching search in another database and that did not answer the research question.

**Figure 1:**
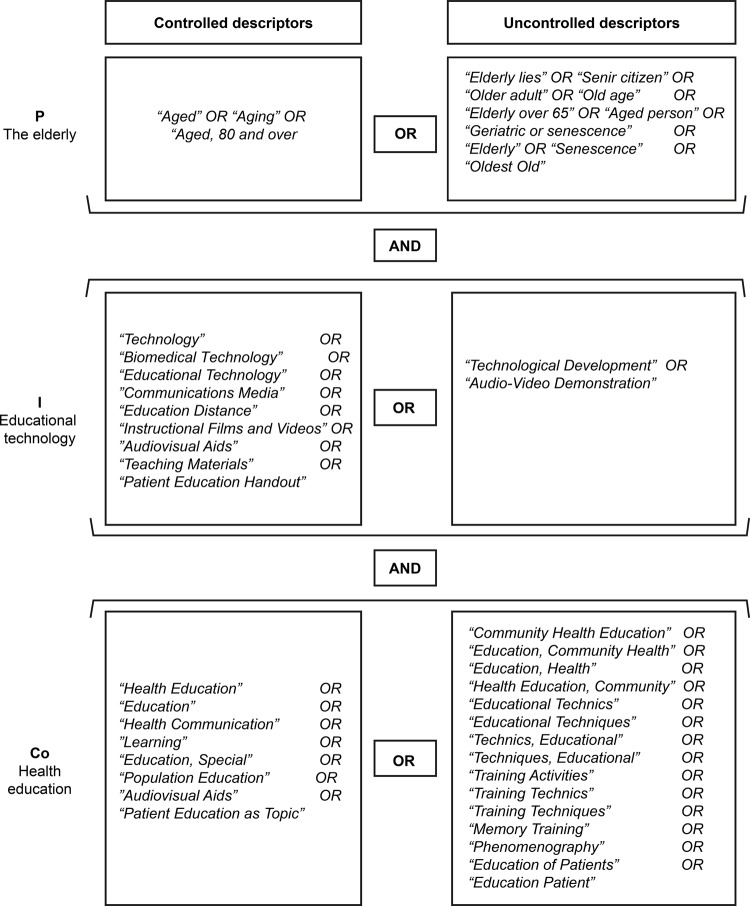
– Controlled and uncontrolled descriptors employed in the search strategy for population, intervention and results. Teresina, PI, Brazil, 2018

For the database search, there was selection of descriptors present on the Health Sciences Descriptors (DeCS) and their English-language equivalents found on the *Medical Subject Headings* (MeSH) and CINAHL titles, as well as selection of uncontrolled descriptors, established according to controlled descriptor synonyms and by means of previous readings on the topic of interest. In order to systematize the sample collection, the advanced search form was used and the peculiarities and characteristics of each database were respected. The descriptors were combined with each other by means of the Boolean connector OR, within each set of terms of PICo strategy, and then crossed with the Boolean connector AND, as shown in Figure 1.

The search was conducted by two independent researchers, who simultaneously standardized the sequence of use of the descriptors and crossings in each database, and then compared the results obtained. In order to guarantee a broad search, the *papers*, in their entirety, were accessed through the journal portal of the Coordination and Improvement of Higher Level - or Education - Personnel (CAPES), in an area with *Internet Protocol* (IP) recognized at the *Universidade Federal do Piauí.*

The studies found were imported into Endnote Web bibliographic reference management software, available in Web of Science database to order the studies found and identify the duplicates in the different databases. This software takes into account database order of exporting and creation of their folders on the file manager, thus selecting the most recent study included as a duplicate. It should be emphasized that exporting articles prioritized the specific nursing (CINAHL) and health (MEDLINE/Pubmed; LILACS; Cochrane) databases, followed by nonspecific ones (Web of Science; Science direct; Scopus).

An instrument adapted from the Form of the *Red de Enfermería em Salud Ocupacional* - RedENSO Internacional (12) was used for extraction and synthesis of information related to the selected studies. The following pieces of information were extracted: year of publication, country, journal, authors’ professional category, study design, theoretical reference used, study objective, educational technology, and outcome.

The level of evidence was determined according to the classification: level I - meta-analysis of controlled and randomized studies; level II - experimental study; level III - quasi-experimental study; level IV - descriptive/non-experimental study or qualitative approach; level V - case report or experience; level VI - expert consensus and opinion^13^.

**Figure 2:**
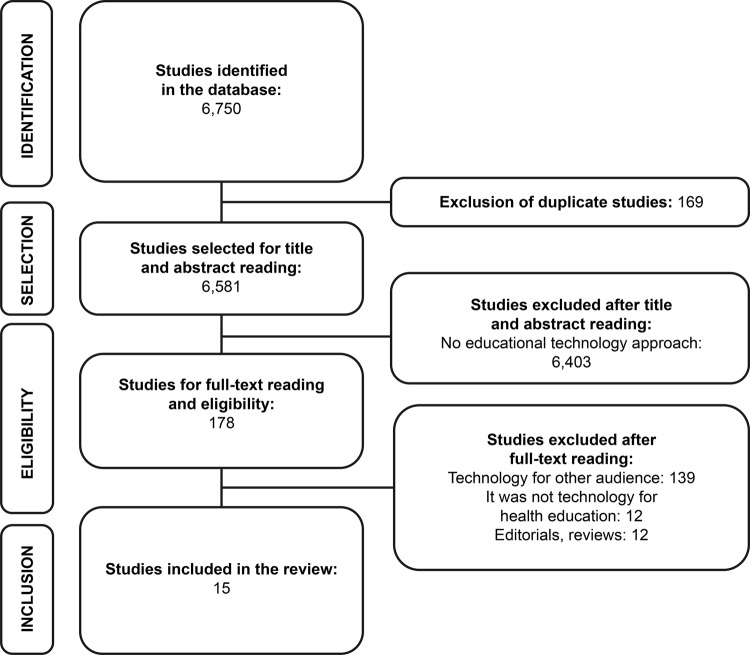
– Flowchart for the selection of primary studies, based on PRISMA recommendation*(14). Teresina, PI, Brazil, 2018

^*^PRISMA =^*Preferred Reporting Items for Systematic Reviews and Meta-Analyses*^

There was identification of 6,750 publications, and 15 articles were selected for the review sample after inclusion and exclusion criteria application. No other studies were included after the manual search process. For publication selection, the recommendations of the *Preferred Reporting Items for Systematic Reviews and Meta-Analyses* (PRISMA)^14^ were followed, as presented in Figure 2.

Critical analysis and qualitative synthesis of the selected studies were performed descriptively in five analytical categories according to the types of educational technologies identified: “software”; “video”; “printed material”; “mock-up”; and “telephone support.”

Since it was an integrative review, the research was not submitted to the Research Ethics Committee, but the ideas of the authors of the publications used in the development of this study were maintained.

## Results

In this review, 15 articles were selected, where one (6.7%) was identified on MEDLINE/Pubmed, eight (53.3%) on CINAHL, one (6.7%) on Web of Science, and five (33.3%) on Cochrane. Regarding these 15 articles, six (40.0%) were published on nursing journals, six (40.0%) on interdisciplinary health journals, and three (20.0%) on journals in other health areas (psychology, medicine and occupational therapy).

All articles included were written in the English language. Regarding the authors’ professional category, four (26.6%) articles were written by doctors, three (20.0%) by doctors in partnership with nurses, two (13.3%) by nurses, one (7%) by architects, one (6.7%) by psychologists, one (6.7%) by communication designers in partnership with nurses, and one (6.7%) by nutritionist in partnership with information technologists. It was not possible to identify this information in two (13.3%) publications.

With regard to the study design, eight (60.0%) were experiments, three (20.0%) were methodological studies, two (13.3%) were quasi-experimental studies, and one (6.7%) had a qualitative approach. As to the level of evidence, nine (60.0%) publications were classified as level II, four (26.7%) as level IV, and two (13.3%) as level III.

Regarding the topics covered by the educational technologies, it was observed that falls in the elderly were focused by five (33.3%) studies and drug treatment by two (13.3%). Each of the following subjects was addressed by one study: cognitive load, self-management of health issues, communication of the deaf elderly, end of life, nutritional education, stoma care, diabetes *mellitus* and HIV/AIDS.

Of the 15 primary studies included, only five (33.3%) were based on the construction/development of educational technology in different theoretical references: Cognitive Theory of Multimedia Learning; Health Belief Model; Situated Learning Theory; Complexity Theory; and Behavioral Cognitive Approach.

The studies were divided into five categories, according to the type of educational technology developed. However, two studies fit into more than one category because they were pieces of research that tested the effects of different technologies.

**Figure 3:**
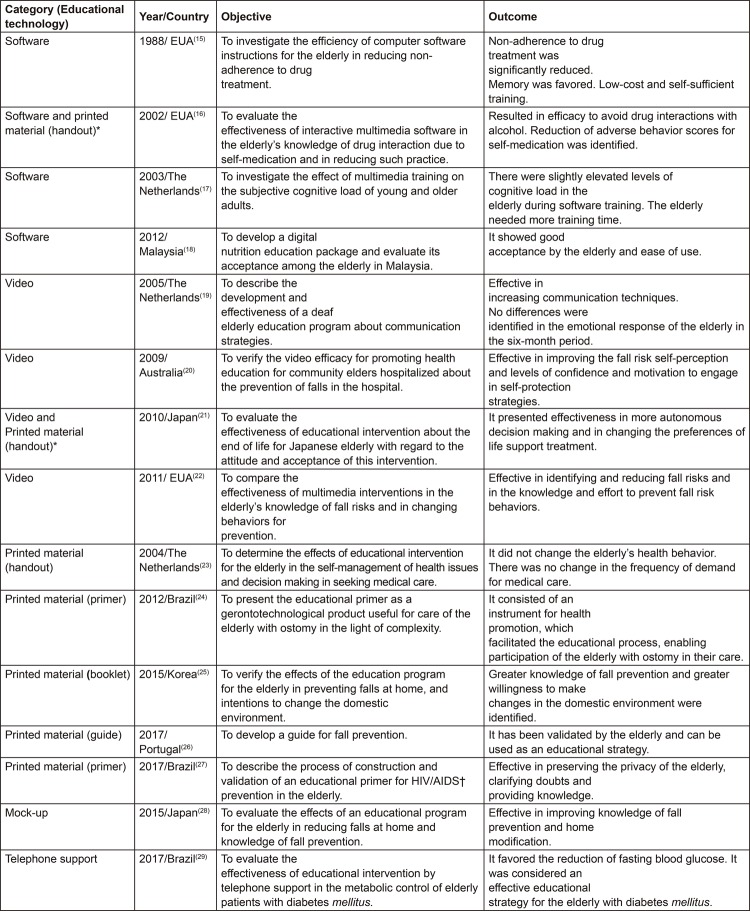
Summary of review articles according to the objective of the study, educational technology, and outcome. Teresina, PI, Brazil, 2018

^*^Study included in more than one category; †HIV/AIDS = Human Immunodeficiency Virus/Acquired Immunodeficiency Syndrome

Figure 3 presents the types of educational technologies developed to promote health education for the community elderly, as well as the objectives and outcome of each study.

The characteristics of the development of the technologies and the interventions implemented in the studies are presented below.

Software is in the first category and appears in the studies as manipulation of the technology on computers and touchscreen equipment by the elderly^15-18^. Among the studies belonging to this category, two studies tested individually their effects by comparison with standard interventions or by the use of the handout, and had their effects evaluated after a two-week follow-up^15-16^.In turn, a primary study compared the effects of technology on the elderly and young adults^17^. The process of software development and evaluation by the elderly was described in only one primary study^18^.

The results of the studies included in this category demonstrated positive effects with regard to the improvement of the outcomes tested. Nevertheless, the authors pointed out that the interventions presented required previous training of the elderly to use the equipment, in addition to a certain degree of schooling for manipulation.

The second category deals with the use of video in individual interventions^19-22^. Among the studies, three have used this technology in different ways, namely: a series of short films^19^, as part of an educational program^21^ or associated with a motivational strategy^22^. Regarding the device used for storage, only two presented this information: one study used videotape^19^, and in another, the device used was DVD^20^. With the exception of one study^20^, the other studies verified the effects of video application in different follow-up periods: two evaluated the outcome from one month of intervention^21-22^, and another from six months of intervention^19^.

The results showed that this type of educational technology was effective in improving the learning of different subjects by the community elders.

Regarding the third category, printed material, the seven studies included developed and/or applied educational technology as booklet, primer and guide, which transmit information through written text and pictures^16,21,23-27^. Among these, three primary investigations were carried out with the objective of constructing and validating the technology^24,26-27^. The others evaluated effects of the use of the material on intervention research, with different periods of follow-up before and after material reading by the elderly, that is, two weeks^16^, three weeks^25^, one month^21^ and three months^23^.

Of the four intervention studies included in this category, only one showed greater effectiveness in the individual use of the printed material. It should be noted that this intervention consisted of the use of the booklet by the elderly, with weekly reinforcement before outcome evaluation^25^.

In the fourth category, the mock-up was presented as a small scale representation of a given structure. Only one intervention study used this technology, which was employed in group educational activity at just one moment. The individual effects were evaluated at 12 and 52 weeks after mock-up presentation. The researchers identified that there was a progressive improvement of the outcome studied^28^.

The fifth category included telephone support, used in research that carried out the monitoring of the elderly health through 16 telephone calls, with educational content, for four months. The results indicated an improvement in the outcomes studied after this period^29^.

## Discussion

This literature review revealed that the technologies developed to promote health education for the community elderly were mainly software, videos and printed materials, but other types of technologies were identified. Thus, it is noticed that the recognition of the rapid growth of the elderly population worldwide has stimulated researchers in the production of multiple technologies to promote health education for this population. However, more investment is needed in the construction and evaluation of these materials in order to increase the possibilities of interventions for clinical practice.

It was identified that among the articles included in this review, the production of the first technology to promote health education for the community elderly was in 1988^15^ Nevertheless, it has been observed that in the 21^st^ Century the production of such technologies has grown gradually, especially between 2012 and 2017^18,27^. It is believed that the expansion of knowledge and dissemination of methods of construction and validation of educational materials have contributed to the technological innovation in the area of gerontology. Therefore, in this perspective, the addition of new scientific productions is expected in the coming years.

It is verified that the articles found were developed in different countries, with emphasis on the United States, the Netherlands and Brazil. However, it is noteworthy the lack of scientific production on the subject of this study in African continent, although it is the second most populous in the world and with high population aging level^30^. In Brazil, this production can be justified, because the elderly health is a priority defined in the National Agenda of Priorities in Health Research, supported by the National Policy for Science, Technology and Innovation in Health, which brings the development of gerontotechnologies as a strategy for health^31-32^.

Nursing journals were the main sources of dissemination of the knowledge produced on educational technologies for the elderly, although the authors were from different areas. It is understood that these results meet the role of nursing in promoting health through educational actions.

The analysis of the articles allows pointing out a knowledge gap in the development of educational technologies that contemplate the multiple gerontological aspects. Nonetheless, the researchers’ investment in the development of fall prevention technologies is understood, since it is a global public health problem that has a direct impact on the health sector^33^. In Spain, 13.9% of the elderly who suffered a fall had to seek health care^34^; in Canada, this prevalence was 5.8%^35^, and in Brazil, 7.8%^36^. The magnitude of this problem goes beyond its frequency, as the consequences can lead to functional disability. However, there are other important health demands in this population, making it necessary the development of educational technologies that consider the multidimensionality of the elderly.

The predominance of randomized trials as study designs demonstrates methodological rigor in the development of technologies for health education for the elderly. Studies of this nature are relevant to the health systems and clinical practice of nursing, as it has the potential to explain cause and effect of different interventions. This result shows the interest in the production of consistent scientific evidence and supports the practical use of the technologies presented here from EBP perspective.

The development of educational technologies, guided by theoretical references, allows the use of concepts and principles that enhance the achievement of the expected educational objective^37^. The analysis of the primary studies, included in this review, points out fragility in the theoretical basis of researches that developed and evaluated the effects of educational technologies on the community elderly, since only a third of the studies used theories to support this process. Therefore, it is necessary that the researchers disclose and explain the theoretical foundations that support the construction and/or application of technology that aims to contribute to the practice.

In addition, among the studies that mentioned the theoretical reference, it was observed the foundation on theories of distinct areas of knowledge. This is possibly due to the fact that the interdisciplinarity of health care extends the fields of theoretical foundation in research, especially in gerontology. Thus, the emphasis is on the perspectives of contribution to and valuation on the nursing science, through the recognition and application of its theories in the construction of educational technologies for the elderly.

All studies included in this review used at least one type of educational technology as an instrument for the educational care process with community elders in order to contribute to meaningful health education.

Among these, it is observed that software-type technology contributes to health education because, through visual, tactile and auditory stimuli, it exercises memory and helps retention of information^38^. However, in order to increase the effectiveness of interventions with this type of educational technology, it is important to emphasize that the differences in the educational profile of the elderly residents in developed and developing countries, such as Brazil, should be considered, since the low rate of elderly literacy is still a reality, which should be considered in the planning of new technologies for health education for this population^39^. Thus, prior training and supervision of the correct use of this type of technology become challenging.

The awakening to the use of software by the elderly favors the rupture of the paradigm of the digital exclusion of this population, through their active involvement in the manipulation of such technologies and construction of autonomous learning. Individual interventions also encourage self-care and adoption of behaviors that promote active and healthy aging and are in line with health education.

The video was another technology present in the studies. It allows the use of several simultaneous and playful resources and favors the construction of mental images or visual association, enabling learning, memorization and construction of specific skills^40^. All the studies included in this review, using this type of technology, indicated its effectiveness in the intervention^19-22^. Among these, it was also noticed its use associated with other teaching strategies^21^. Other studies have found positive effects in teaching different subjects to other populations mediated by video^41-43^. Thus, the adequacy of this type of educational technology as a health education strategy isolated or associated with other technologies is verified.

Despite understanding the advantages of video for health education, it is noted that few studies have developed this material for the elderly. Therefore, investments of gerontology researchers in the construction, validation and evaluation of the effects of educational videos for the elderly are paramount.

The printed material was presented as the type of educational technology most developed for the community elderly. In addition, it was observed that the same study may have been classified in more than one analytical category, since two studies, included in the other categories, compared the effectiveness of those technologies with a printed material (handout)^16,21^. However, among those which tested the effects of this type of technology alone, only one study that used the booklet in an educational strategy with weekly reinforcement, achieved the results expected^25^.

It was also noticed that three studies presented the construction and validation of primers and guide^24,26-27^. It is therefore recognized that it is important to make this type of material available for use in health services. However, the need for testing the effect of the use of such materials by community elders, through randomized controlled trials, is emphasized

This type of technology offers the elderly the opportunity of autonomy for the study on a certain theme, with the possibility of reinforcement and quick access, and encourages self-responsibility about the own health. For this, the Functional Health Literacy (FHL) is considered a pathway that favors the promotion of elderly health, since it means the capacity to obtain, process and understand health information, aiming at self-management in health^44^.

In Brazil, a study that evaluated the FHL conditions of diabetic elderly found that 73.7% had low FHL, and this was associated with schooling^45^. It is understood, therefore, that educational printed materials for the elderly should be used with caution and take into account the simple and objective language, which favors the correct understanding of the information.

Another technology found in the primary studies was the mock-up, which was effective in guiding a realistic understanding of the changes in home fall risk factors in a Japanese population. In addition, it was observed that there was retention of knowledge after different follow-up periods^28^.

With regard to health education, the use of the mock-up seems to provide an interactive practice, making realistic three-dimensional observation and manipulation a therapeutic moment for the development of skills and knowledge for long-term decision making. The mock-up becomes, therefore, a new possibility of interaction of theoretical information with practice, intensifying the educational care. However, there is need of further scientific investigation of the effects in the implementation of this technology on other outcomes for application in geriatrics.

In relation to telephone support, its use emerges as an educational technology to enable the expansion of communication and contribute to care. This type of technology is presented as an option for health interventions, complementing standard care.

The incorporation of telephone support into health care is innovative and offers the professional the opportunity to better approximate and follow the health decisions taken by the elderly. In addition, regarding the community elderly, it becomes an effective strategy to address a greater number of people who have difficulties to access health services, whether geographical or financial^46^

Results of an integrative review of the literature indicated positive effects on adult population teaching after the use of telephone support^47^. Therefore, positive value should be added to professional telephone counseling for the community elderly, as it favors a therapeutic and trust relationship between the professional and the service user.

It is also added that telephone follow-up is a nursing intervention established by Nursing Interventions Classifications (NIC)^48^. Thus, since this nursing intervention and its effectiveness contributes to health care, the relevance in the development of other studies that use this technology for health education for the elderly is emphasized.

After analyzing the five categories, we observed the different types of educational technologies presented by the studies included in this review. One realizes the researchers’ interest in incorporating strategies to facilitate the retention of information that allows the improvement of different aspects of the elderly health.

The development of these technologies for the community elderly should respect their educational needs, as well as seeking to meet their expectations. For this purpose, one should consider cross-cultural care and popular education, since the elderly’s way of thinking and acting comes from the context in which they live^49^. This promotes greater interest in the use of educational technology and involvement in the health education process.

It was found that among the experimental studies included in this review, the effectiveness of the technologies was tested in different contexts and time intervals between the intervention and outcome evaluation. In this way, new studies can be developed with application and measurement of the effects of the use of these materials at different moments and outcomes.

Regarding the approach used by the study researchers to evaluate the effects of the technology application, the individual presentation of the materials stood out, to the detriment of the group intervention, found only in one study^28^. It is thus identified that the use of such technologies in elderly groups should be tested in new studies, since it is the main approach used in educational actions in public health, and that it enhances autonomy and empowerment through closer therapeutic ties and knowledge exchange among the actors involved in this approach^50^.

It should be emphasized that each technology has its importance within the context of health education and it is up to the nursing professional, in partnership with patients and others involved in the care process, to choose the ones that best fit the social reality of the community elderly. In addition, the use of educational technology should not reduce assistance procedures to simple techniques, but rather strengthen relationships, facilitate dialogue, humanize care and effectively promote health.

As a limitation of this review, the inclusion of only studies that have developed educational technologies for the community elderly is indicated, thus not considering technologies for institutionalized elderly, which restricts the results for the use of such technologies in this public.

## Conclusion

It was identified, in this integrative review, that there were several technologies developed for the community elderly health education. Printed materials, software and videos prevailed, but there was also identification of mock-up and telephone support. Fall prevention was the topic most addressed by the studies, whose majority performed an experiment and verified the effectiveness in the individual use of the educational technologies to promote health education for community elderly.

The following knowledge gaps are indicated: little number of elderly health area themes addressed by educational technologies; fragile theoretical foundation regarding the studies on technologies development; lack of research on this review subject in the African continent, and limited amount of studies that combined the use of different educational technologies, testing their use in elderly groups. Performance of other studies that develop educational technologies for the elderly on different gerontology topics is suggested. In addition, theirs effects on health education with different approaches should be tested through longitudinal trials in order to evaluate long-term effects.
